# Quantitative–Qualitative Assessment of Dream Reports in Schizophrenia and Their Correlations with Illness Severity

**DOI:** 10.3390/brainsci14060568

**Published:** 2024-06-03

**Authors:** Gianluca Ficca, Oreste De Rosa, Davide Giangrande, Tommaso Mazzei, Salvatore Marzolo, Benedetta Albinni, Alessia Coppola, Alessio Lustro, Francesca Conte

**Affiliations:** 1 Department of Psychology, University of Campania “L. Vanvitelli”, 81100 Caserta, Italy; gianluca.ficca@unicampania.it (G.F.); oreste.derosa@unicampania.it (O.D.R.); davide.giangrande@studenti.unicampania.it (D.G.); tommaso.mazzei@studenti.unicampania.it (T.M.); benedettaalbinni@gmail.com (B.A.); alessiacoppola001@gmail.com (A.C.); alessio.lustro@studenti.unicampania.it (A.L.); 2 Dermatology Unit, Department of Mental and Physical Health and Preventive Medicine, University of Campania “L. Vanvitelli”, 80138 Naples, Italy; 3 Residential Community for Therapy and Rehabilitation “Al di là dei sogni (Beyond Dreams)”, 81037 Sessa Aurunca, Italy; salvatore.marzolo@aslcaserta.it; 4 Mental Health Unit 15, Local Health Authority 1, 81016 Piedimonte Matese, Italy

**Keywords:** dreams, schizophrenia, emotion regulation

## Abstract

Positive symptoms of schizophrenia have been proposed to be an intrusion of dreaming in wakefulness; conversely, psychotic patients’ abnormal cognitive and behavioral features could overflow into sleep, so that their dreams would differ from those of healthy people. Here we assess this hypothesis by comparing dream features of 46 patients affected by schizophrenic spectrum disorders to those of 28 healthy controls. In patients, we also investigated correlations of dream variables with symptom severity and verbal fluency. Overall, patients reported fewer and shorter dreams, with a general impoverishment of content (including characters, settings, interactions) and higher spatiotemporal bizarreness. The number of emotions, mainly negative ones, was lower in patients’ reports and correlated inversely with symptom severity. Verbal fluency correlated positively with dream report length and negatively with perceptive bizarreness. In conclusion, our data show a significant impoverishment of dream reports in psychotic patients versus controls. Future research should investigate to what extent this profile of results depends on impaired verbal fluency or on impaired mechanisms of dream generation in this population. Moreover, in line with theories on the role of dreaming in emotion regulation, our data suggest that this function could be impaired in psychoses and related to symptom severity.

## 1. Introduction

“I had an historical dream! I was at Berlinguer’s funeral. There was this Pertini character trying to escape by bike, I chased him.”

Patient n.17

Schizophrenia is a psychotic disorder characterized by disturbances in cognition, emotional responsiveness and behavior. According to the first criterion for the diagnosis of schizophrenia in the DSM-5 [1], at least two of the following symptoms must be present for most of the time for at least one month: (1) delusions; (2) hallucinations; (3) disorganized speech; (4) grossly disorganized or catatonic behavior; and (5) negative symptoms such as reduced volition or emotional expression. Several phenomenological similarities have often been underlined between dream features and these symptoms (e.g., [2]). As reviewed in Limosani et al. [3], besides sensory perceptions in absence of external stimulations which are shared by dreams and psychosis (i.e., hallucinations), cognition is characterized, in both states, by disorganized thought and unrealistic ideational contents, accompanied by bizarre experiences for which the subject shows an impairment of reality testing and, subjectively, a very intense emotional involvement. 

These similarities clearly bear implications for both psychopathology and research on dream processes. Indeed, it has been suggested that dreams may represent a natural model for psychosis (e.g., [4]) and that schizophrenia could be a sort of “trapped state” between waking and dreaming, with the encroachment of experiences usually occurring in dreams into wakefulness [5]. For dream researchers, however, the dream–psychosis relationship is also extremely interesting the other way round, i.e., addressing the influence of wakefulness on sleep mentation and the possibility of making predictions on psychotic patients’ dreams given the peculiar characteristics of their disorder. As a matter of fact, there are solid research lines trying to understand whether and to what extent waking experience is reflected in dreaming, in line with the widely held “continuity hypothesis” according to which a continuity would exist between waking-life experiences and dreams [6].

Furthermore, the relationships between wakefulness and dreams would be reflected not only in dream content, but also in its associated emotions [7]. Notably, a primary function of sleep for emotion regulation has been repeatedly proposed (e.g., [8,9]), also in light of REM sleep’s peculiar neurotransmitter balance, which is believed to provide optimal conditions for offline processing of affects (see [10] for a review). Our own group has recently shown that poor sleep quality might impair sleep-related processes of affect regulation [11]. Thus, we deem it extremely interesting to look at dream features in the disturbances of the schizophrenic spectrum, where emotional dysregulation is a key characteristic.

According to what was said so far, waking-life psychotic symptoms could be directly linked to specific dream characteristics. When coming to schizophrenia spectrum disorders, this would imply that psychiatric symptoms could be predictors of differences in the dreams of psychotic patients relative to those of controls. However, probably due to the methodological difficulties of collecting dream reports from this kind of patient, the data available on this topic are still very sparse and foggy.

Dream reports in schizophrenia were found to be shorter in a number of rather old studies (e.g., [12,13,14]), but their results were obtained with different methodologies and did not clarify whether they were accounted for by an actual reduction in dream generation or by patients’ reduced ability to recall and report their dreams, e.g., due to the impairment of verbal fluency repeatedly shown in schizophrenia [15].

Concerning their qualitative features, schizophrenic patients’ dream reports, compared to healthy controls’, were occasionally displaying reduced emotional involvement and emotional expression [16,17], less affect and less change in dream scenery [18], more frequent presence of familiar people [19] or of strangers [20,21], fewer words referring to the semantic field of hearing and a less active role of the dreamer [17].

Cognitive bizarreness—defined as “impossibility or improbability in the domains of dream plot, cognition and affect” [22]—has been considered as a cognitive marker shared by psychotic waking and dreaming state, but to what extent the high bizarreness in schizophrenic patients’ waking ideation is maintained during dreams is still an open issue. Early studies found less bizarreness in schizophrenic patients’ dreams in comparison to the dreams of a normal control population [12,23,24], whereas the more recent literature seems to point to equal [21] or even higher bizarreness scores in schizophrenic patients [25]. Interestingly, a study by Scarone et al. [26] showed that a comparable degree of formal cognitive bizarreness was shared by the waking cognition of schizophrenic subjects and the dream reports of both normal controls and schizophrenics. 

In sum, the sparse and contrasting literature does not allow to draw clear conclusions on the relationships between dream features and psychosis. Therefore, here we compare dream reports of schizophrenic patients to those of healthy controls with regard both to quantity (Dream Recall Frequency, from now on DRF) and quality (length, content), in order to provide further data to enlighten the issue of whether psychotic symptomatology is reflected in dream content. Within the frame of this general objective, we specifically intend to focus on a few issues that have been covered very little, if at all, by previous research: (a) the possible role of lexical access ability, indexed by verbal fluency performance, in affecting dream report length; (b) the amount and types of emotions reported in dreams in the clinical vs. the control group; (c) the relationship of dream features with illness severity, as measured through the Brief Psychiatric Rating Scale (from now on, BPRS).

## 2. Materials and Methods

### 2.1. Participants

We recruited a convenience sample of 46 patients with diagnosed psychotic symptoms (F 10, M 36, age range: 19–54 years) at a residential facility (Rehabilitation Community “Beyond dreams”, Sessa Aurunca (Caserta), Italy, n = 26) and a day-treatment center (“Integrazioni”, Casoria (Napoli), Italy, n = 20) and 28 volunteer healthy participants (F 17, M 11, age range: 20–59 years), who were psychology students of the University of Campania “L. Vanvitelli” (Caserta, Italy) and their relatives and friends. 

The main inclusion criterion for the patients’ group (PG) was having a diagnosis of schizophrenia or any other psychotic disorder according to DSM-V criteria [1], including schizoaffective disorder. All patients were being treated with combined individual and group integrative psychotherapy. Family therapy was also followed by 24% of the subjects. Pharmacological treatments, which had to have been stable for at least three weeks before the study, were distributed as follows: no medication, 10.9%; mono therapy with antipsychotics, 32.6%; polytherapy with antipsychotics and benzodiazepines (only one patient was treated with zolpidem instead of benzodiazepines), 56.5%. Other inclusion criteria were (a) absence of comorbidity with other psychiatric or neurological disorders; (b) no evidence of mental retardation; (c) for inpatients in the residential facility, having stayed there for not less than 1 month and not more than 18 months.

As for the healthy control group (CG), inclusion criteria were (a) age between 18 and 60; (b) absence of any history of somatic and/or psychiatric disturbances; (c) absence of any history of sleep disorders; (d) regular sleep habits, evaluated through the Pittsburgh Sleep Quality Index, Italian Version [27]. Also, only subjects who reported recalling at least one dream per week were recruited. 

All demographic characteristics of the two samples, including clinical diagnosis, therapies for the patients’ group, are listed in Table 1.

### 2.2. Procedure

The study was approved by the Ethical Committee of the Department of Psychology, University of Campania (Italy). After providing information about the study, consent forms from both patients and healthy controls were obtained.

Before dream report collection, the psychopathological severity of each PG participant was evaluated through the BPRS, Expanded Edition 4.0 [28] during a one-hour individual therapy session. Moreover, a verbal fluency test [29] was administered to the same group to evaluate lexical access ability.

Participants were requested, 5 days a week (over a period of 30 days for the patients’ group and of 15 days for the healthy controls), to report, immediately at spontaneous awakening, the mental activity they had memory of through the following classical instruction (presented in written form): “Please tell me everything you can remember of what was going through your mind before you woke up.” [30]. For patients in the residential facility, it was the facility staff who solicited them to report the dreams at awakening, whereas patients from the day-treatment center and normal controls were instructed to write down or audio-record their dreams first thing after awakening. The patients were also requested to fill in a diary of their daily activities to control that they kept their daily routines stable, without any peculiar experience that might influence their dreams. 

### 2.3. Instruments

For psychotic symptom severity assessment, we administered the BPRS [28], in its Italian version [31]. The BPRS has shown good internal consistency, with a Cronbach’s alpha of 0.87 [32]. The severity of each one of the 24 symptoms is rated on a scale from 1 to 7, ranging from 1 (Absent) to 7 (Extremely severe). Ratings are based both on the patient’s answers to the interviewer’s questions and on the observed behavior during the interview. According to the total BPRS score, psychotic subjects were assigned to one of seven severity groups (Absent 0–24, Very Mild 25–48, Mild 49–72, Moderate 73–96, Moderately severe 97–120, Severe 121–144, Extremely severe 145–168).

The Verbal Fluency Test used in our study [29] consists of two tasks: Semantic fluency and Phonemic fluency. Subjects are given 1 min to produce as many words as possible within three semantic categories (i.e., “Car brands”, “Fruits”, “Animals”) or starting with three given letters (i.e., “P”, “F”, “L”), respectively. Total scores for both tasks correspond to the total number of words generated in each task. These scores are then corrected for age and education in order to obtain equivalent scores from 0 to 4, where 0 is considered “pathological” and 4 “above normal”. The test has shown good internal consistency, with a Cronbach’s alpha of 0.83, and test–retest reliability within acceptable limits (i.e., r = 0.74) [33].

Finally, the diary of daily activities administered to patients consisted of two questions: (1) What were the main activities of your day? (2) What kind of emotions did you experience (positive, neutral, negative)?

### 2.4. Dream Analysis

Dream reports were evaluated by two independent raters, with a third rater, blind to the design and aims of the research, called to resolve possible disagreements. Whenever the subjects claimed that they had made “more than one dream”, if they referred them to different bouts of sleep, only the report of the dream preceding the awakening was taken into account. Otherwise, the different dreams were considered as a single report (as in [34]).

Dream Recall Frequency (DRF) is defined as the percentage of days in which a dream report was obtained over the whole number of days of the protocol.

Length of reports was measured in temporal units (TUs) according to Foulkes and Schmidt’s method [35]. A TU is assigned whenever (a) a character performs an action that, in waking life, could not be performed synchronically with his/her previous action; (b) a character responds to another character or event; (c) there is a topical change in the dream report.

Following Occhionero and Cicogna [36], type of Self-representation was coded into six categories:

(1) Presence of Self as a pure thinking agent;
(2) Total or partial Self body image, more or less associated with proprioceptive, kinesthetic, agreeable or painful sensations;
(3) Representation of Self as a passive observer of the dream events;
(4) A precise hallucination of both mind and body, analogous to wakefulness;
(5) Identification with other characters in the dream;
(6) A double representation of Self with two distinct and relatively active roles.

A seventh category—(0) Absence of Self-representation both as a physical entity and as thinking subjectivity—was added here to describe reports in which it is not possible to identify any type of Self-representation.

A number of dream content dimensions (as in [37]) were analyzed through a set of dichotomous categorical variables (presence/absence of that feature in the dream report):

- Continuity, scored as present when the report’s narrative structure did not show sudden interruptions or changes of main settings or characters (when the report was described as containing more than one dream, no continuity was assigned).
- Impossibility/Implausibility Bizarreness, referring to events whose occurrence is implausible during wake;
- Space/Time Bizarreness, referring to spatiotemporal distortions;
- Perceptive Bizarreness, referring to images, characters or objects with distorted shapes, colors or dimensions;
- Emotions, referring to spontaneously verbalized emotions which are clearly expressed by the subject and felt by the dreamer himself during the dream (other characters’ emotions reported by the dreamer were not included);
- Positive Emotions;
- Negative Emotions;
- Somatic Sensations, referring to spontaneously verbalized somatic sensations clearly expressed by the dreamer;
- Non-Self Characters, referring to any additional character besides the Self;
- Unknown Characters, referring to strangers or unfamiliar characters, appearing as single individuals or undefined groups;
- Interactions, referring to direct (Self) and indirect (Others) interactions between characters;
- Friendly Interactions, referring to friendly direct (Self) and indirect (Others) interactions between characters;
- Aggressive Interactions, referring to aggressive direct (Self) and indirect (Others) interactions between characters;
- Sexual Interactions, referring to sexual direct (Self) and indirect (Others) interactions between characters;
- Setting, referring to a specific, clearly identifiable setting in which the oneiric scene takes place.

Three dream content dimensions were also assessed as continuous variables:

(a) emotions (only those spontaneously verbalized by the subject in the dream report are included in scoring these variables): total number of emotions (including both the dreamer’s and other characters’ emotions), number of Self (dreamer’s) emotions, number of non-Self (other characters’) emotions;
(b) characters: number of characters (these were scored only when they appeared as single individuals: both familiar and unknown characters were included, but the dreamer and undefined groups were excluded);
(c) interactions (all actions identified by verbs clearly referring to interactions): total number of interactions, number of Self-interactions (those between the dreamer and other characters), number of non-Self interactions (those between two or more non-Self characters).

These continuous variables were all analyzed both as absolute numbers and as percentages over the number of TUs. 

### 2.5. Statistics

A Kruskall–Wallis ANOVA was conducted to test between-group (CG vs. PG) differences in dream variables. Except for the analysis of between-group differences in (DRF) and white reports frequency, patients producing 0 dream reports over the whole data collection period were excluded from the analysis (N = 19). Spearman’s correlation coefficient (rr) was used to detect possible correlations between age, global score at the BPRS, verbal fluency (phonemic and semantic), and dream variables in PG. Between-group differences in age and years of education were assessed with the Mann–Whitney U test, whereas differences in gender distribution with the Chi-Square (χ^2^) test.

All analyses were performed with Jamovi 2.3.21 (The Jamovi Project, 2023), and the statistical significance level was set at *p* ≤ 0.05. Descriptive statistics are reported as mean ± standard deviation.

## 3. Results

### 3.1. Characteristics of the Sample

The two groups did not differ in age (total sample: 35.7 ± 10.6; CG: 33.0 ± 6.8; PG: 36.0 ± 11; U = 99.5, *p* = 0.634) but differed in gender (total sample: F 27, M 47; CG: F 17, M 11; PG: F 10, M 36; χ^2^ = 6.81, *p* = 0.009) and years of education (CG: 16.0 ± 2.7; PG: 11.4 ± 2.3, U = 28.0, *p* = 0.003).

More than half of the cases (56.5%) are schizophrenias of the paranoid and disorganized types, whereas the remaining 43.5% is divided between unspecified schizophrenia, schizoaffective disorder, and schizotypic personality disorders. Only 5 patients are drug-free and 26 of them are taking benzodiazepines and/or hypnotic drugs in addition to antipsychotic medications. Table 1 summarizes the patients’ characteristics derived from the initial clinical assessment, namely diagnoses, pharmacotherapies, BPRS score and Verbal Fluency.

### 3.2. Inter-Rater Agreement

Inter-rater agreement turned out to be satisfactorily high both for the analysis of temporal units (r = 0.94) and for that of content variables (r = 0.92).

### 3.3. Diary of Daily Activities of Patients

Overall, patients carried out different activities during the data collection period, the most frequent of which were recreational activities (28%), training–work activities (23%), individual and/or group therapy (19%), meeting with family members (19%), and outdoor activities (11%). The most reported emotions during activities were neutral (50%), followed by negative (30%), and positive (20%).

### 3.4. Dream Recall Frequency

Nineteen patients did not produce any dream report across the entire data collection period (non-recallers: 41.3%). Instead, all the healthy controls produced at least one dream report. A total of 159 dream reports were obtained from the patients’ group over the 30-day study period while controls’ dream reports (collected over a 15-day period) were 111 overall. Therefore, DRF in the patients’ group was significantly lower than in controls (18% ± 1.33 vs. 27% ± 4.25, χ^2^_1_ = 9.30, *p* = 0.002). In PG, non-recallers did not differ from recallers in age, years of education, and gender distribution, but significantly differed in BPRS global score, reporting higher severity of psychopathological symptoms (Table 2). 

DRF did not significantly differ between inpatients (21.61 ± 1.71) and patients treated at the day-care facility (i.e., sleeping at home) (13.23 ± 2.26; χ^2^_1_ = 3.8, *p* = 0.060). 

A significantly higher proportion of contentless dreams, i.e., “white reports”, was reported by patients compared with controls (PG: 4.00 ± 0.40 vs. CG: 0.00 ± 0.00, χ^2^_1_ = 8.12, *p* = 0.004).

### 3.5. Between-Group Differences in Dream Report Features

Overall, PG reported significantly shorter dream reports than CG, and dream report length was higher in the inpatients group (2.70 ± 1.20) relative to the day-care facility patients (1.62 ± 0.77; χ^2^_1_ = 4.48, *p* = 0.034).

**Figure 1:**
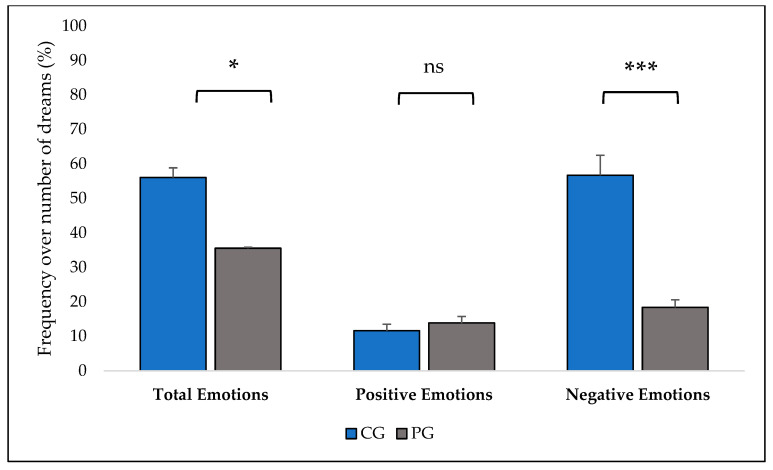
Differences between healthy controls (CG) and patients (PG) in total, positive and negative emotion frequency. *: *p* < 0.05; ***: *p* < 0.001; ns: not significant.

Compared with CG, dream reports in PG were characterized by significantly fewer emotions (Table 3, Figure 1), fewer non-Self, unknown, and total characters, fewer interactions (all types except sexual ones), and a lower frequency of a precise setting. Furthermore, PG reported higher space/time bizarreness (see Table 3 for the complete results). 

The two groups did not differ in the type of Self-representation in dreams (Table 4).

### 3.6. Associations of Dream Variables with Symptom Severity and Verbal Fluency in the Patient Group

The BPRS global score was positively correlated with the frequency of non-Self characters in dreams (*p* = 0.028) and negatively with the number of emotions (*p* = 0.030).

**Figure 2:**
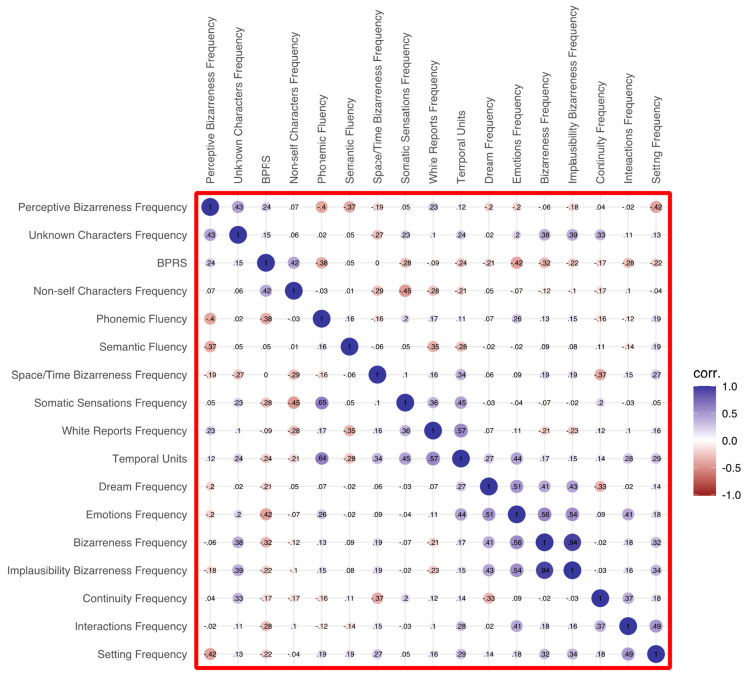
Heatmap of the correlations in the patient group.

As for verbal fluency, phonemic fluency positively correlated with dream report length (i.e., temporal units; *p* = 0.040) and frequency of somatic sensations (*p* = 0.037). Furthermore, both phonemic and semantic fluency were negatively correlated with the frequency of perceptive bizarreness (*p* = 0.036 and *p* = 0.011 respectively). The full heatmap of correlations is depicted in Figure 2.

## 4. Discussion

To the best of our knowledge, this is one of the very few studies analyzing the dream characteristics of a relatively wide sample of individuals with psychotic symptoms (compared to healthy subjects) in their habitual life context over an extended period of time (30 days) and assessing the relationships of their dream features with their illness severity and lexical ability. 

As for quantitative aspects, our data are consistent with previous findings [12,20,23] showing a decreased frequency of dream recall in individuals suffering from psychotic disorders compared with healthy subjects. Additionally, dream reports appeared shorter in this group, which is also in agreement with a number of past results [12,13,14,26]. When interpreting these data, it is always very difficult to understand to what extent they depend on reduced oneiric production or, instead, on lower efficiency of dream retrieval processes in schizophrenic subjects. This is a well-known issue in all populations who show a similar quantitative reduction in dreams, such as the elderly [38]. Here, the overall low DRF in the patient population is largely accounted for by the rather high percentage of complete non-recallers, dramatically decreasing the DRF value, which would otherwise appear similar between patients and controls. On the other hand, the correlation between verbal fluency and dream report length, as well as the higher proportion of contentless reports (those mental activities the subjects are aware of but whose content they are unable to verbalize, also called “white reports”) observed in the patient sample, support the hypothesis of an impairment in the process of verbally reporting dreams, whereas any conclusion on a possible reduction of oneiric production per se remains speculative. This idea is also coherent with our finding that non-recallers of the patient group showed significantly higher BPRS scores than recallers, suggesting that higher severity is accompanied by greater difficulties in dream recall processes.

Concerning dream content, a consistent profile emerges of global poverty in patients’ dreams relative to those of healthy subjects. In the patients’ reports, there are fewer characters, fewer settings, a lower number of total interactions, accounted for by the reduction of friendly and aggressive ones, both referring to first-person interactions or to other people’s interactions witnessed as an observer. This profile suggests that dreams in psychotic patients are less rich and articulate than those of controls; however, again, considering that verbal fluency influenced dream report length, we cannot exclude that the scarcer contents of patients’ dreams depend simply on their deficient lexical access abilities. As for bizarreness, it is not surprising that it was not found to be higher in PG, except for an increase of the space-time type. In line with this, as in Scarone et al. [26], we did not find any correlation between the BPRS severity score and dream bizarreness in the patient group. As suggested by the same authors [26], the gap between patients and controls in bizarre cognition during wakefulness appears to be filled during dreams. 

One of the core results of this study refers to the rather impoverished emotional pattern of patients, with a decreased average number of emotions (paralleled by a lower number of reports in which emotions are expressed). Strikingly, this result, which is concordant with previous data [16], almost totally depends on the significant reduction of negative emotions. Apparently, the classical phenomenon observed in healthy subjects of negative dream emotions prevailing over positive ones [39,40] appears inverted in psychotic individuals. This phenomenon in healthy subjects has been interpreted as a possible index of the role of dreams in emotion regulation [7,11,41,42]: in fact, the negative emotions experienced more frequently or intensely in the period in which the dream occurs would be those in need of regulation during sleep, whereas positive emotions, requiring less modulation, would be underrepresented in the dream [7,11]; alternatively, the phenomenon could be due to a rebound of thoughts and related emotions suppressed during wakefulness [41,42]. Therefore, our result in the patient group comes as an impressive counterpart of their severe emotional dysregulation during wakefulness and warrants further investigations, e.g., in terms of its magnitude as a function of illness severity, given that, in our sample, we found that the number of dream emotions decreased as a function of illness severity. 

In this regard, the negative association that emerged between BPRS scores and emotions is in line with data from Schredl and Engelhardt [43], who found correlations between the scores on the Symptom Checklist-90-R scales and dream features in patients with several mental disorders, including schizophrenia. These data suggest that it is the severity of specific symptoms (such as depressive mood or psychotic symptoms), rather than the diagnostic classification, that is primarily related to dream content. 

Another interesting finding is the lack of differences between CG and PG in the representation of Self and one’s own body. This feature has been occasionally described in healthy subjects [36,44,45], with data pointing to the presence of changes in the representation of the Self according to age and the sleep state from which the dream is recalled (being more similar to wakefulness in REM sleep dreams and more polymorphous in NREM sleep dreams). To our knowledge, this is the first time that Self-representation is assessed in a clinical population. Even in this aspect, there appear to be no particularly atypical characteristics transposed from wake symptomatology to the dreaming experience. The same can be observed for the frequency of somatic sensations, which also does not differentiate PG from CG.

A final speculation should be reserved to the issue of whether the present findings are in favor of the “continuity hypothesis” [6], whose original formulation might be usefully reported here: “*(…) dreams are continuous with waking life; the world of dreaming and the world of waking are one. The dream world is neither discontinuous nor inverse in its relationship to the conscious world. We remain the same person, the same personality with the same characteristics, and the same basic beliefs and convictions whether awake or asleep. The wishes and fears that determine our actions and thoughts in everyday life also determine what we will dream about)*”. On a theoretical level, the hypothesis of continuity between waking and dream features seems particularly suited to the psychotic population in light of the similarity of this population’s cognitive, emotional and behavioral characteristics during wakefulness with those of dreams (e.g., sensory perceptions in the absence of external stimulations, disorganized thought, unrealistic ideational contents, intense emotional involvement). In addition, these phenomena appear to be based on common neuroanatomical and neurochemical mechanisms of the waking and dreaming states. For instance, vivid sensory and motor imagery is related to a specific kind of neurotransmission imbalance, with lowered serotoninergic and noradrenergic tone relative to an increase of the cholinergic one [4]; disorganized thoughts are probably linked to the drastic reduction of functional brain connectivity, involving the majority of brain areas in dreams [46] and the thalamocortical circuits in schizophrenia [47], whereas bizarre experiences and the absence of reality testing are most likely dependent on a reduction of frontal cortex activity, especially in the dorsolateral areas [48]. Finally, the physiological basis for emotional involvement could be the remarkable activation of the amygdala and other limbic areas, clearly shown by functional neuroanatomy in both REM sleep [46,49] and schizophrenia [50]. 

However, it still seems hazardous to draw conclusions on the continuity hypothesis in psychotic individuals in a straightforward manner. In our study, the massive misperceptions, hallucinations, bizarre and disorganized thoughts and behaviors that are commonly associated with the psychotic experience do not seem to emerge in dreaming, at least not more pronouncedly than in healthy subjects. Actually, greater continuity appears to emerge with regard to the impoverishment of dream content, which parallels negative rather than positive psychotic symptomatology. Indeed, the manifestations of psychoses, especially in patients who have a long history of the disease and are chronically medicated, are often characterized by a prevalence of cognitive symptoms, such as remarkable deficits in working memory and attention [51]. Therefore, to what extent the impoverished dream reports found in our sample are a result of “continuity” with waking clinical features remains an open issue, which could prompt, in the future, the characterization of patients in a more refined way, e.g., through the use of instruments assessing day-by-day symptomatology in the positive, negative and cognitive dimensions. In addition, the correlation discussed above between verbal fluency measures and dream report length does not allow the ruling out of the possibility that greater continuity between waking and dreaming features would emerge if lexical access were not impaired. 

Importantly, this same observation has to be kept in mind when interpreting our data (as well as any other data on dreaming in psychotic populations) in light of current theories on dreaming and psychosis. Specifically, our data do not appear to be compatible with the recent Defensive Activation Theory [52], which posits that REM sleep dreams and positive neurological symptoms play a “defensive” role against the rapid expansion of functional brain areas over understimulated ones (the visual cortex during sleep and functionally impaired networks in neurological syndromes) [53]. According to this hypothesis, we should have observed, in the dreams of the psychotic sample, a richness of elements even greater than that of healthy controls, reflecting the activation of a double protective process, i.e., that exerted by REM sleep dreaming for the benefit of the visual cortex (as in controls) and that exerted by more general overstimulation processes (reflected in positive symptomatology during wakefulness) in favor of wider brain networks impaired by psychiatric pathology. Instead, our data are coherent with another recent theoretical account, which interprets psychotic symptoms in light of the Active Inference Theory [54]: specifically, positive symptoms are considered as an attempt of the central nervous system to overcome the mismatch between predictions and outcomes occurring when top-down attentional processes generate expectations but not predictions of their content [53]. As for negative symptoms, they have been interpreted as arising from uncertainty in social prediction brain circuits [55]: the general dearth of elements in our patients’ dreams, as well as specifically the paucity of characters, interactions and emotions, could be seen as reflecting this kind of mechanism.

The findings in this study have to be interpreted cautiously in light of a few limitations. The first regards a crucial methodological choice, i.e., collecting dream reports at spontaneous awakening in the participants’ habitual living environment. This approach is somewhere between the high experimental rigor achieved through report collection at provoked awakenings in the lab—which is, however, an extremely difficult procedure for clinical samples as demanding and delicate to deal with as psychotic patients—and retrospective interviews, much easier to collect but very unreliable in terms of waking interference and content bias [21]. Therefore, here experimental control is partially sacrificed in favor of an ecological design, determining both meaningful advantages and limitations. On the limitations side, it was not possible to control for the actual adherence of the participants sleeping at home (control subjects and the patients at the day care center) to the instruction of reporting their dream immediately at awakening. Also, we could not collect prompted reports, which would have solicited dream recall with more completeness than solely spontaneous reports, allowing us to better clarify the role of memory retrieval impairments in the scarcity and poorness of dream reports. Moreover, we could not obtain objective sleep data, making it impossible to relate dream report variables to the sleep states during which the dream was produced. On the other hand, our study design permits ruling out the insertion of a non-familiar environment in dreams (see the interesting discussion on that in [56]), since patients’ and controls’ habitual sleep habits and environments were respected. Furthermore, although our sample size is limited and participants were selected based on geographical proximity and accessibility, our research protocol allowed us to reach a sample of patients that is quite wide compared with most of the literature on the topic, as well as to obtain a fairly extended collection period (several weeks), which would have been scarcely feasible with a laboratory design (this usefully enlarged the total number of dream transcripts despite the low expected DRF). 

Another limitation regards the disparity we found between groups in gender proportion and years of education. Regarding the latter, the difference was expected since educational attainment is generally lower in schizophrenia [57], probably due to the fact that the age of onset for this kind of pathology peaks around 20 years. As for the gender differences, studies on this topic consistently show that these differences mainly involve dream thematic content (e.g., sexuality, aggressive behaviors, etc.), whereas length, realism and level of interaction are similar [58,59]. Therefore, we are confident that the gender disparity between groups did not significantly affect our data, since our main result essentially regarded a general impoverishment of patients’ dreams, affecting most of the dream elements. 

Furthermore, it must be taken into account that most participants in the patient group were being treated with psychotropic medications (mostly antipsychotics, but also benzodiazepines and hypnotics), which have been shown to have some effect on dream features (especially DRF, although data are sparse and conflicting) [60]. 

Finally, for the patient group, although we collected and analyzed BPRS scores, we lack precise information on the presence and severity of positive and negative symptoms, which could have been useful to discuss our findings in light of recent theories on dreaming and psychosis. However, as mentioned above, because of the influence of impaired verbal fluency on dream report production, any interpretation in accordance with these theories would still have been speculative. 

## 5. Conclusions

In conclusion, our data show significant quantitative and qualitative differences between dream reports of psychotic patients and those of healthy controls, collected over extended periods of time (several weeks) in the participants’ habitual living environments. Specifically, patients show lower dream frequency as well as shorter dream reports. Moreover, healthy participants’ dreams appear much richer and more articulate in terms of several content aspects, including number of characters, settings, interactions and emotions. The lower amount of negative emotions reported by patients and the correlation found between number of emotions and illness severity scores are in line with theories on the role of dreaming in emotion regulation, suggesting that this function could be impaired in psychoses and related to symptom severity. Finally, our data appear compatible with current theoretical accounts on dreaming and psychosis (such as the continuity hypothesis [6] or the Active Inference Theory [54]). Further research conducted within the framework of these hypotheses is warranted to investigate to what extent the general impoverishment of psychotic patients’ dreams depends on their impaired verbal fluency or on impaired mechanisms of dream generation. 

## Figures and Tables

**Table 1:** Clinical features in the patients’ group.

Variable		
Diagnosis (N, %)		
	Paranoid Schizophrenia	16 (34.80)
	Disorganized Schizophrenia	10 (21.70)
	Schizotypic Personality Disorder	8 (17.40)
	Unspecified Schizophrenia	6 (13.00)
	Schizoaffective Disorder	6 (13.00)
Pharmacotherapy (N, %)		
	No Drugs	5 (10.90)
	Monotherapies	15 (32.60)
	Politherapies	26 (56.50)
Brief Psychiatric Rating Scale Total Score (m ± sd)		82.19 ± 20.46
BPRS Severity groups (N, %)	Absent	0 (0)
	Mild	5 (10.90)
	Moderate	10 (21.70)
	Moderately severe	18 (39.10)
	Severe	11 (23.90)
	Very severe	2 (4.30)
	Extremely severe	0 (0)
Verbal Fluency		
	Phonemic (m ± sd)	5.84 ± 3.81
	Semantic (m ± sd)	11.7 ± 3.75

**Table 2:** Characteristics of dream recaller and non-recaller patients.

Variable	Recallers	Non Recallers	Statistics
Age	35.30 ± 10.83	37.10 ± 11.36	U = 235, *p* = 0.631
Sex	22 M 5 F	14 M 5 F	χ^2^_1_ = 0.4, *p* = 0.528
Years of education	11.2 ± 2.06	11.7 ± 2.56	U = 213, *p* = 0.288
BPRS	77.10 ± 21.30	89.40 ± 17.26	U = 162, *p* = 0.036 *

Notes. Significant *p*-values are marked with an asterisk.

**Table 3:** Distribution of dream content features in the two groups.

Variable	CG	PG	χ^2^_1_	*p*
Temporal Units (n)	4.85 ± 2.35	2.43 ± 1.17	3.56	<0.0001 *
Continuity (%)	75.95 ± 8.50	84.28 ± 2.47	1.56	0.211
Bizarreness (%)	38.54 ± 2.56	41.45 ± 3.13	0.00	0.952
Implausibility Bizarreness (%)	33.75 ± 2.61	37.80 ± 3.34	0.02	0.898
Space/Time Bizarreness (%)	7.29 ± 1.55	14.13 ± 1.53	5.31	0.021 *
Perceptive Bizarreness (%)	11.90 ± 1.76	7.63 ± 1.26	0.53	0.468
Emotions Frequency in Dreams (%)	56.03 ± 2.84	35.52 ± 0.30	5.76	0.016 *
Emotions (n)	0.85 ± 0.42	0.42 ± 0.39	12.49	<0.001 *
Positive Emotions (%)	11.61 ± 1.86	13.85 ± 1.93	0.47	0.492
Negative Emotions (%)	58.68 ± 5.82	18.39 ± 2.18	17.56	<0.001 *
Somatic Sensations (%)	15.00 ± 2.14	15.77 ± 1.82	0.33	0.562
Non-Self Characters (%)	95.71 ± 0.94	82.21 ± 1.82	10.74	0.001 *
Unknown Characters (%)	73.00 ± 2.46	36.39 ± 3.24	15.00	<0.001 *
Characters (n)	1.98 ± 0.73	0.71 ± 0.65	24.81	<0.001 *
Interactions (%)	94.46 ± 1.20	52.79 ± 3.33	25.65	<0.001 *
Interactions (n)	1.89 ± 0.70	0.71 ± 0.49	29.46	<0.001 *
Interactions Self (n)	1.45 ± 0.55	0.61 ± 0.41	25.75	<0.001 *
Interactions Others (n)	0.45 ± 0.30	0.09 ± 0.28	23.59	<0.001 *
Self-Friendly Interactions (%)	66.71 ± 2.26	32.37 ± 2.72	17.22	<0.001 *
Self-Aggressive Interactions (%)	24.05 ± 2.20	6.76 ± 1.37	11.35	<0.001 *
Self-Sexual Interactions (%)	2.67 ± 0.78	3.51 ± 0.93	0.16	0.684
Others Friendly Interactions (%)	16.19 ± 1.87	2.55 ± 0.53	10.02	0.002 *
Others Aggressive Interactions (%)	15.98 ± 1.71	2.25 ± 0.98	11.81	<0.001 *
Others Sexual Interactions (%)	2.68 ± 1.04	0.46 ± 0.02	0.36	0.549
Settings (%)	88.06 ± 1.77	53.42 ± 3.48	15.21	<0.001 *

Notes. Frequencies are expressed as the percentage of dreams in which the feature was present over the total number of dreams obtained from that group. CG: Control Group, PG: Patient Group. Significant *p*-values are marked with an asterisk.

**Table 4:** Frequency distribution of different types of Self-representation in the two groups.

	Type 0	Type 1	Type 2	Type 3	Type 4	Type 5	Type 6
CG	2%	1%	1%	1%	90%	1%	1%
PG	7%	0%	1%	3%	87%	1%	1%
χ^2^_1_	1.96	0.30	1.87	0.01	0.96	0.00	0.84
*p*	0.161	0.578	0.172	0.895	0.326	0.959	0.359

Notes. CG: Control Group; PG: Patient Group.

## Data Availability

The original contributions presented in the study are included in the article; further inquiries can be directed to the corresponding author due to privacy reasons.
